# Diffusion of a Lifelog-Based Digital Healthcare Platform for Future Precision Medicine: Data Provision and Verification Study

**DOI:** 10.3390/jpm12050803

**Published:** 2022-05-16

**Authors:** Kyuhee Lee, Jinhyong Lee, Sangwon Hwang, Youngtae Kim, Yeongjae Lee, Erdenebayar Urtnasan, Sang Baek Koh, Hyun Youk

**Affiliations:** 1 Artificial Intelligence Big Data Medical Center, Wonju College of Medicine, Yonsei University, Wonju 26417, Korea; powerpc@yonsei.ac.kr (K.L.); arsenal@yonsei.ac.kr (S.H.); edenbyra@yonsei.ac.kr (E.U.); 2 Lifelog Bigdata Platform Business Group, Wonju College of Medicine, Yonsei University, Wonju 26417, Korea; hahae0618@yonsei.ac.kr (J.L.); getmuk@yonsei.ac.kr (Y.K.); yjlee9977@gmail.com (Y.L.); 3 Department of Preventive Medicine, Wonju College of Medicine, Yonsei University, Wonju 26417, Korea; kohhj@yonsei.ac.kr; 4 Department of Emergency Medicine, Wonju College of Medicine, Yonsei University, Wonju 26417, Korea

**Keywords:** digital healthcare, precision medicine, healthcare platform, diffusion of digital healthcare

## Abstract

We propose a method for data provision, validation, and service expansion for the spread of a lifelog-based digital healthcare platform. The platform is an operational cloud-based platform, implemented in 2020, that has launched a tool that can validate and de-identify personal information in a data acquisition system dedicated to a center. The data acquired by the platform can be processed into products of statistical analysis and artificial intelligence (AI)-based deep learning modules. Application programming interfaces (APIs) have been developed to open data and can be linked in a programmatic manner. As a standardized policy, a series of procedures were performed from data collection to external sharing. The proposed platform collected 321.42 GB of data for 146 types of data. The reliability and consistency of the data were evaluated by an information system audit institution, with a defects ratio of approximately 0.03%. We presented definitions and examples of APIs developed in 17 functional units for data opening. In addition, the suitability of the de-identification tool was confirmed by evaluating the reduced risk of re-identification using quasi-identifiers. We presented specific methods for data verification, personal information de-identification, and service provision to ensure the sustainability of future digital healthcare platforms for precision medicine. The platform can contribute to the diffusion of the platform by linking data with external organizations and research environments in safe zones based on data reliability.

## 1. Introduction

Lifelogs are real-world data of daily lives recorded and stored on personal devices, portable storage systems, or in the cloud. Lifelogging involves a series of procedures that collect and process data through sensors and smart devices [1,2,3,4,5,6,7,8]. A personal health record is considered a dataset consisting of an individual’s lifelog as well as a hospital’s clinical data. The combination of lifelog and clinical data in digital healthcare services is beneficial and powerful for precision medicine; therefore, lifelogs are becoming a new research trend that can improve the quality of daily life and expand insights based on big data analyses of individuals’ daily activities and health records [9,10,11,12]. However, there are limitations in using them as digital healthcare services, because lifelogs and clinical results are collected individually in organizations and hospitals.

With the aging population, the number of people suffering from chronic diseases is increasing and expenditure has increased to manage them [13]. Although expectations for high-quality medical services are rising, the burden of personal medical expenses continues to expand owing to population decline and low economic growth. Statistically, in the case of young hypertensive patients aged 20–39 years, the recognition, treatment, and control rate of hypertension were found to be very low compared with other age groups [14]. In the case of diabetes, 20.9% of diabetic patients need active treatment with glycated hemoglobin of 8.0% or higher, and only 8.4% of them have their blood sugar, blood pressure, and cholesterol under control [15,16]. In Korea, in 2017, chronic obstructive pulmonary disease (COPD) had a high prevalence of 13.3% in adults over 40 and 28.3% in those over 65. With 12.9 deaths per 10,000 people, it is the 8th leading cause of death worldwide [17]. To manage these chronic diseases on a full-cycle basis, it is necessary to build an integrated platform that includes the patient’s medical information as well as the lifelog, by establishing a big data statistical analysis and life-cycle management system.

The effects of lifestyle on chronic diseases have been studied in various ways. Ontology methods that can be used to integrate heterogeneous smart devices have been studied to collect lifelogs generated in individual lives [18,19,20,21,22]. Ontology-based research defined the range of lifelog data, identified and classified concepts, and performed a comparative evaluation using a similarity index. These lifelogs can be used meaningfully in connection with the concept of the personal health record (PHR), which was introduced by Carl Dragstedt (in 1956) in the U.S. [23]. Currently, many hospitals have introduced EHR and EMR systems to perform patient care and hospital management. Big data based on EMR and EHR are being built and operated as platforms to provide services to patients and researchers [24]. Europe and Australia have already applied the clinical data generated in hospitals to big data platforms for digital healthcare [25]; however, healthcare platforms focus only on connecting hospitals and individuals based on a hospital’s medical information; therefore, lifelogs produced by individuals and medical information produced by hospitals are fragmented, which limits the provision of better medical services to patients and high-quality data to researchers [26,27,28,29,30]. In addition, for the diffusion of digital healthcare platforms, the quality of data must be assured through purification and consistency verification. We have already developed a concept and proof for big data-based platforms that utilize lifestyle and medical information, and they are operational [1].

In this study, we verify the data quality and service of the platform and propose a method to secure reliability of precision medicine in future digital healthcare. The tools developed by the platform for pseudonymization or anonymization were verified according to the national de-identification guidelines [31,32], so that individuals can provide information with confidence. The proposed lifelog-based digital healthcare platform (LDHP) is aimed at providing high-quality precision medical services to individuals with chronic diseases by analyzing lifelogs and clinical information. The LDHP supports raw data, statistical analysis, and AI-based deep learning engines that are useful for researchers and organizations. In addition, for the diffusion of the platform, we present a system that analyzes related data with the national data map of Korea [33]. APIs developed for easy utilization of the platform’s data and services can help create a virtuous cycle ecosystem by providing flexibility in data utilization to companies and researchers.

## 2. Materials and Methods

**Figure 1:**
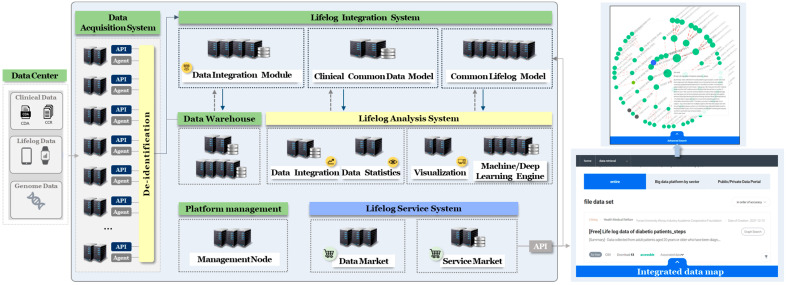
Lifelog-based digital healthcare platform.

The proposed digital healthcare platform is a cloud-based system for collecting, analyzing, sharing data, and providing statistical analysis and AI-based deep learning of medical information and lifelogs. The platform consists of five components: a data acquisition system (DAS) that collects, refines, and transmits lifelogs and medical information to the platform; a lifelog integration system (LIS) for data processing and management; a lifelog analysis system (LAS) that stores and analyzes processed data; it provides deep learning based on AI and visualization; and a lifelog service system (LSS) that provides data distribution and services. Centers preprocess data based on a predefined data catalog or column definition and they load it into the cloud space. Subsequently, personal information is de-identified before being sent to the platform and sent to the data warehouse (DW) of the platform through the developed API or agent. The transmitted data are processed into products that can be opened through statistical analysis or AI-based learning, and can be found in the data or service markets. The LDHP is shown in Figure 1.

### 2.1. Data Centers

The LDHP consists of a consortium of 13 centers producing lifelogs and clinical data. Five medical centers produce data from clinical outcomes and eight lifelog data centers produce data based on daily lifelogs, such as walking, weight, nutrition, and activities. All data centers are cloud-based and implemented using private infrastructure.

Medical data center: Data centers based on clinical data warehousing produce clinical data. The centers use cohort data from long-term follow-up surveys to identify health conditions to determine the causal association between risk factors and disease outbreaks and to include medical information related to chronic diseases. In addition to pre-established clinical data, lifelogs have been collected using wearable devices in clinical trials.

Lifelog data center: Centers comprising healthcare startups produce health data such as blood sugar and blood pressure from individual lifelogs and certified medical devices. Lifelogs include data from walking, nutrition, exercise, weight, and surrounding environment information. After data cleansing, these data are transmitted to the local server of each center, and then the DAS of the platform.

### 2.2. Data Acquisition System

In Figure 1, the DAS is a private cloud space that is connected to the local space of the centers. It has increased security where only administrators of the center can access it using SSL-VPN. When the center loads data in the DAS, the queue method is adopted to ensure smooth operation, even in the event of a service interruption owing to an error. Centers in the DAS transmit data to the platform using an API or agent after data validation and de-identification of personal information. Data transmission is processed periodically; however, a retransmission function is provided in the case of data or system errors. Data management is implemented so that the target of deletion can be easily identified by setting the storage period and the total amount of original storage.

The center manager verifies the validity of the collected data based on a predefined data catalog and schema before sending it to the DAS. If the validation fails, a log is generated in units of its fields or records; however, if validation is successful, the center manager de-identifies personal information. We designed repositories by applying standardization to data (structured, semi-structured, and unstructured), and it was distributed and stored in file systems, RDBMS and NoSQL, depending on the data type. The platform has developed and provided a GUI-based data upload program to easily handle the above procedures.

When data validation is successful, the center manager transmits it to the platform using the cryptographic hash algorithm SHA-256/512 to ensure the integrity of the data that does not contain personal information. If the data contain personal information, they are pseudonymized or anonymized using a developed de-identification tool. In 1996, the Health Insurance Portability and Accountability Act (HIPAA) was enacted in the United States to standardize the electronic exchange of medical-related administrative and financial data [31]. In September 2020, the Republic of Korea revised and announced the guidelines for the safe use of healthcare data [32]. De-identification of personal information is based on the privacy protection model set by the platform; however, it may also be modified by the center administrator. After de-identification is completed, the center manager must obtain approval from the review committee, composing of information security experts, to determine its suitability.

### 2.3. Data Analysis System

The LAS provides AI-based deep learning and statistical analysis tools. It is implemented with representative open sources, such as R-Studio, Zeppelin, and Jupyter. LAS can analyze lifelog and clinical data stored in DW using a statistical package. The analyzed results are visualized such that the end user can easily interpret them. Analytical and raw data stored in a DW can be customized for customers through machine learning or deep learning. In terms of the diffusion of data and services aimed at the platform, it has the advantage of opening analysis data and AI-based deep-learning modules tailored to startups or researchers who suffer from technological shortcomings. We operate online/offline safe zones for customized services. These safe zones were set as the platform’s demilitarized zones to strengthen security. Researchers can use statistical and artificial intelligence tools in the online safe zone to process the desired data and export them as statistical or deep learning data.

### 2.4. Data Warehouse

The data validated from DAS are finally re-checked for quality using database-based quality management tools installed in the DW of the platform. The DW is a core element of the platform as it stores lifelogs and clinical outcomes as raw data, processes them into the desired dataset, and stores them. The DW communicates organically with the platform components and stores the results processed in each module. For example, a module can result in a statistical analysis, API service, machine learning/deep learning engine, or visualization. The platform sells data products to consumers in a metadata-based market using files or APIs for a fee or free-of-charge.

LDHP established rules for metadata verification and history management. For example, consumers can use metadata to filter and identify items necessary for the actual data material, owner, description, quality, security information, historical information, and utilization analysis. Using such metadata, the platform enables a semantic search through natural language processing. In addition, various medical information and lifelogs on the platform help researchers derive meaningful results by considering the correlation between the data. The meta-management system in DW not only makes it easy to use the data needed for processing, but it also supports additional processing in the statistical and AI-based deep learning engine of LAS.

### 2.5. Lifelog Service System

The data processed and fused in the integrated system of the platform are stored in DW and then reprocessed and provided as data products in the LAS. LSS is divided into two markets to provide processed datasets and services. The data market sells processed or analyzed products and the service market provides innovative services and APIs to check users’ health information. The dataset consists of several products in one package.

#### 2.5.1. Data Provision

LDHP provides datasets for a fee or free-of-charge through the data market. All data are anonymized to protect privacy. To download a dataset, a research plan including IRB approval should be submitted, and then the data manager of the platform decides it according to the decision of the data review committee. In addition to direct downloads via the data market, it can be downloaded through an app or in a programmatic manner using APIs. For downloads using the API, if the data administrator of the platform approves it, an authentication token is issued and can only be downloaded by the authorized user.

For the diffusion of the digital healthcare platform, we established a national data-sharing system in connection with the integrated data map. Researchers can use the API on a data map or download it through a direct link of the platform’s data product. In both cases, the platform checks the log information for the statistical analysis of downloads. We have developed 17 types of APIs for each functional unit to manage the products. Table 1 provides an API definition for product searches and an example of field usage.

#### 2.5.2. Service Provision

We launched four innovative services that allow anyone to check their health information by entering parameters (age, weight, underlying disease, blood pressure, blood sugar, and BMI) for public opening and platform diffusion. The four innovative services are blood sugar management evaluation, electrocardiogram-based blood component prediction, comorbidity prediction for individuals with diabetes, and cardiovascular disease prediction for individuals with metabolic syndrome. Online and offline safe zones are operated with enhanced security to prevent leakage of sensitive information when using data analysis services. In the two safe zones, big data-based statistical analysis of lifelogs and clinical data is possible, and researchers can generate their desired model using an AI-based deep learning engine. Researchers with limited knowledge of big data statistical analysis or AI can solve problems with technical help from experts on the platform.

The sequence of using the safe zone is as follows:

1. Submission of research plan for data analysis;
2. Check the security pledge and procedures in the control area;
3. Approval from information protection manager of the platform;
4. Utilization of user’s safety zone;
5. Security verification for data export;
6. Data export.

### 2.6. Policies

The LDHP established a full-cycle management policy, from data collection to operation, utilization, and disposal. The data lifecycle management policy is applied to all data on the platform and depending on the characteristics, deletion and backup policies were included. The operational policy of the data and service markets is applied differently depending on the supply method. Free products are supplied with anonymized original data; if processing is required, the actual cost is charged. Paid products comply with “Lifelog-based digital healthcare platform terms and use” and “Data transaction support guidelines” of KDATA (Korea Data Agency) [34]. The security policies include technical security and privacy protection. The platform was implemented on a cloud system certified by ISO/IEC 27,799 for medical data storage and CSAP for cloud security certification [35,36,37,38,39,40].

## 3. Results

The validity of the lifelog and clinical data is verified during the loading of data and de-identifying personal information. The loaded data are additionally verified for consistency and validity by the information systems audit institution annually. In this section, we describe the status of the produced data and the method for data opening. In addition, we analyze the consistency and error rate evaluated by the information system audit institution and verify the tool for the de-identification of personal information.

### 3.1. Data Production

**Figure 2:**
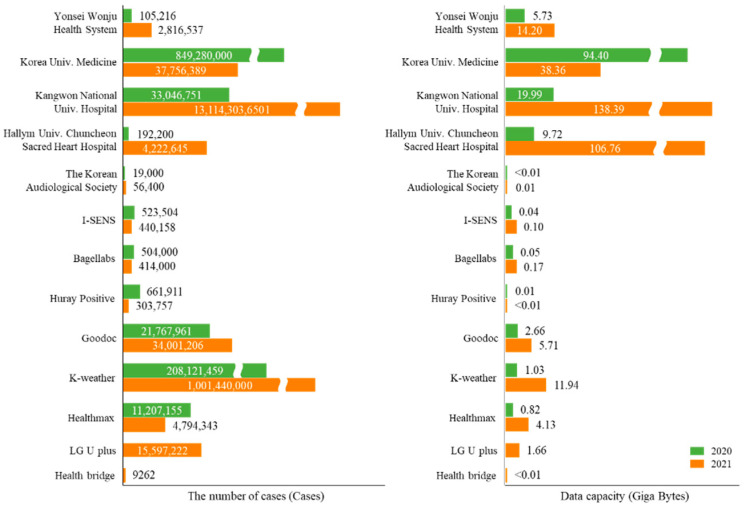
Data contribution in cases and capacity.

We obtained clinical data and lifelogs from 13 data centers. The dataset collected in the first year was updated in the second year, and 52 new datasets were produced. We divided the produced datasets into medical data centers and lifelog data centers, which are described in Table 2 and Table 3, respectively. In 2020, 11 centers collected about 1.12 billion cases for 94 types of approximately 135.45 GB of data. In 2021, two new data centers were added, collecting about 14.2 billion cases for 156 types of approximately 321.42 GB data. In general, medical data centers had a higher number and capacity of data loaded on the platform than lifelog data centers. The cases and capacities of the data produced in 2020 and 2021 are described in Figure 2 for each center.

### 3.2. Data Validation

The data collected by the platform are evaluated annually for quality certification by the information system audit institution led by the National Information Society Agency in Korea. The information system audit institution evaluates the database of an audited organization for data quality certification. Currently, it is based on the domain and business rules of the audited organization for all factors affecting quality. Quality certification is evaluated according to the guidelines defined by the KDATA. The information system audit institution evaluated the data consistency, referential integrity, and entity integrity using a verification tool on the dataset loaded into PostgreSQL. As a result, we obtained the defects ratio of the data and Six-Sigma, which is a quality management methodology developed by Motorola, Inc. in 1986 [41]. This approach uses data-driven reviews to limit mistakes or defects in enterprises and business processes [42,43]. Moreover, Six-sigma, a six-standard-deviation event from the mean, is required for a mathematical error.

To obtain Six-Sigma, we evaluate the defects per opportunity (DPO), and defects per million opportunities (DPMO), of data stored on the platform [44]. The calculations for DPO and DPMO are as follows:

(1) $$\mathrm{DPO}=\frac{\sum^{\;}n_{errs}}{\sum^{\;}m_{oppts}}$$

(2) DPMO=DPO×1,000,000

In Equation (1), $n_{errs}$ is the number of defects and $m_{oppts}$ is the number of opportunities. The DPMO in Equation (2) was calculated using Equation (1).

(3) Six Sigma=1.5×NORMSINV(1−DPMO/1,000,000)

Finally, we obtain the Six-Sigma value using Equation (3) based on Equation (2). The NORMSINV function in Microsoft Excel calculates the value that proves the standard cumulative normal distribution function using specified mean and standard deviation values. 

The evaluation results calculated using the three equations are listed in Table 4. The defects ratio was approximately 0.01% in 2020 and 0.03% in 2021, and the Six-Sigma value obtained was 5.17 in 2020 and 4.91 in 2021, respectively. The certification grades defined by KDATA in Korea are classified into Silver, Gold, and Platinum classes: The Silver class (Six-Sigma and data consistency ratio are higher than 3.2 and 95.51%); the Gold class (Six-Sigma and the data consistency ratio are higher than 3.5 and 97.70%); the Platinum class (Six-Sigma and the data consistency ratio are higher than 5.0 and 99.97%). In the certification grade of data quality, we were rated Platinum in 2020 and Gold in 2021.

For the de-identification of personal information, we classified data parameters into identifiers and quasi-identifiers according to the platform’s policy. In general, blood test values are de-identified by designating them as quasi-identifiers, because individuals can be implicitly re-identified according to the combination of data by reflecting individual health characteristics. In the privacy protection model, k-anonymity (k = 4) was applied according to the country’s guidelines for the de-identification of personal information [32]. Quasi-identifiers were created by specifying l-diversity (l = 5) and t-closeness (t = 0.2). We used data from the Yonsei Wonju Health System for the de-identification of personal information, which was used only to verify the accuracy and re-identification risk for the prediction of the de-identification tool launched on the platform. In addition, it provides the same re-identification risk as ARX [45], which is the most widely used open-source tool. Table 5 shows the results of the analysis of each parameter for cardiovascular disease-related blood tests in the ARIRANG cohort [46,47] and the re-identification risk before and after de-identification. In Table 5, patient IDs are encrypted, and identifiers such as names and social security numbers are completely anonymized or removed during ETL so that individuals cannot be recognized. As with ARX, the platform’s built-in de-identification tool supports three attacker models (inspection, journalist, and marketer) for re-identification risk analysis. As shown in Table 5, the risk of re-identification is dramatically reduced when de-identification is performed on most of the items. Although WBC has the highest level of 33.33% in the highest risk, it is also significantly decreased compared with the previous level.

### 3.3. Data Provision

To download the dataset, users can click a link directly on the platform or use an API. We developed 17 APIs for product creation, search, information change, file attachment, download, and deletion. The APIs developed for data management and opening are designed based on the example of the definition presented in Table 1 and are summarized in Table 6. In Table 6, the first column describes the API name and communication method, the second column indicates the description, the third column shows the URL used as the API, and the last column shows examples of the outputs that can be received by calling the API. The API’s URL is marked as “Platform Domain”, owing to the platform’s security policy.

## 4. Discussion

In this study, we verified the reliability of the LDHP based on medical information and individual lifelogs. The methods presented for the spread of the platform included specific procedures for data verification, personal information protection, and service provisions. LDHP is operated as a big data platform, collecting 135.45 GB of 95 types in 2020 and 321.42 GB of 156 types in 2021.

In a previous study, a healthcare platform that collected big data using APIs from wearable devices was proposed [19]. In addition, Suciu et al. implemented a semantic big data platform to analyze and visualize heterogeneous wearable data; however, there were no standardized guidelines for collecting and refining data in various institutions [20]. Mano et al. provided a secure smart healthcare monitoring and notification system that processes and analyzes big data to obtain value information [21]; however, this study did not suggest methods to protect personal information such as de-identification. In addition, previous studies have limitations in expanding the digital healthcare platform as a system that individually supports data verification, provision methods, and de-identification, rather than an integrated system. To solve this problem, we presented standardized policies and methodologies for data collection, purification, and de-identification of personal information that can be applied by DAS and LIS centers. In accordance with the presented standard guidelines, each center can perform improved data quality management and verification.

Data consistency and validity were assessed by the center manager using the developed tool, and data consistency, error rate, and certification grade were verified by the information system auditor annually. We obtained defects ratios of 0.01% in 2020 and 0.03% in 2021; therefore, the reliability of the platform’s data quality is very high. In addition, we established guidelines and procedures for protecting personal information based on the platform’s policy and launched a de-identification tool that can be used in DAS. Even in the absence of identifiers in an individual’s medical records and lifelogs contained in clinical information, the risk of re-identification was dramatically reduced by designating it as a quasi-identifier and applying de-identification.

The proposed platform is a new digital healthcare platform that expands the power of information of existing clinical data to daily lifelogs for future precision medicine. To spread this platform to various researchers and companies, we presented a method for providing data. Raw data are basically implemented so that they can be exported through the APIs after personal information is pseudonymized or anonymized. Our platform visualizes relevant public data in connection with a national data map for analysis. The safe zone operated by the platform contributes to its spread by providing researchers with statistical analysis and an artificial intelligence learning environment.

The online/offline safe zone provides an analysis environment and data to researchers and healthcare companies. Researchers who have difficulties in obtaining medical information or establishing an environment can conduct their desired research with only a simple predefined procedure. In particular, startups can derive more accurate analysis results with clinical and technical support from a group of experts on the platform. In addition, it is easy to obtain the types of data that can be analyzed, along with medical data, from an integrated data map operated by the country.

We provide innovative services to predict health status so that individuals can recognize the risk of chronic diseases, and actively treat them. By using the innovative service, individuals can reduce medical expenses and improve their quality of life. As a result, the reduction in individual medical expenses leads to a reduction in the national social cost of public health.

The limitation of this study is that it has only partially been applied to medical information to increase interoperability because it is difficult to directly apply a standardized method such as HL7 to a lifelog; therefore, it is necessary to study how to apply the messaging standards presented by HL7 or IHE to real-world data in the future.

## Figures and Tables

**Table 1:** Example of an API for product search.

Type	Field Name	Type	Description	Input
Header	X-CKAN-API-Key	string	Information retrieval with the authentication key	Authentication ID
Parameters	q	string	Inquiry by adding conditions for each column	“fields_name”:value
	fq	list	Applying filters per the column	“fields_name”:value
	sort	string	Sorting the result	“sort”:”score desc, metadata_modified desc”
	rows	Int *^a^	The number of rows in the query result	The number of lists to be displayed
	start	int	The page in the result	The page number to be displayed
	include_private	bool	Whether to retrieve private datasets	“include_private”:true
	use_default_schema	bool	Use of the default schema	“use_default_schema”:true
	include_drafts	bool	retrieval of draft data	“include_drafts”:true
Result	success	bool	Success or failure of API call	res[“success”]
	result	Dict *^b^	Retrieved results	res[“result”]
	result.count	int	The number of data in the result	res[“result”][“count”]
	result.search_facets	dict	The number of retrieved information by conditions	res[“result”][“search_facets”]
	result.result	int	The item list in result	res[“result”][“result”]

*a: integer, *b: dictionary (data structure).

**Table 2:** The dataset list of medical data centers.

Data Centers	Data Sources	2020		2021	
		Cases	Capacity (GB)	Cases	Capacity (GB)
Yonsei Wonju Health System	Metabolic syndrome’s lifelog	53,210	4.50	67,695	7.76
	12-lead ECG	40,642	1.23	2,541,855	1.90
	Cohort study	11,364	<0.01	82,635	0.01
	Diabetic patient’s lifelog	-	-	123,194	4.52
	COPD patient’s lifelog	-	-	358	0.04
	Integration data	-	-	800	<0.01
Korea University Medicine	CDM data	849,210,000	94.40	36,251,389	38.33
	inPHR data	70,000	<0.01	-	-
	CDM extension data	-	-	1,505,000	0.02
Kangwon National University Hospital	Lifelog data	625,846	0.19	56,639,191	86.39
	Clinical information data	6,683,156	2.00	2,619,241,352	0.24
	Clinical support data	9,179,470	2.80	7,765,402,405	17.98
	Health insurance and other data	16,513,279	5.00	1,483,360,512	27.58
	Clinical and lifelog data of newcomers	40,000	<0.01	369,460	<0.01
	Nutritional images	5000	10.00	25,000	0.07
	Diabetic patient’s lifelog	-	-	138,529	<0.01
	Newcomers’ data	-	-	9,045,808	0.07
	Visit and health checkup data	-	-	511,632	0.05
	Cohort’s clinical data	-	-	1,179,569,762	6.00
Hallym University Chuncheon Sacred Heart Hospital	Smart health data in Kangwon	45,600	2.31	1,390,391	17.93
	Healthy life data in Inje-Yangu	68,500	3.89	1,598,230	28.73
	Healthy life data in Seoul	75,600	2.35	1,202,102	13.70
	Chatbot data for dementia	500	1.17	9277	8.75
	Mild cognitive disorder	-	-	320	0.04
	Telemedicine services	-	-	22,245	37.59
	Dementia data	-	-	80	<0.01
The Korean Audiological Society	Auditory test data	19,000	<0.01	56,400	0.02

**Table 3:** The dataset list of lifelog data centers.

Data Centers	Data Sources	2020		2021	
		Case	Capacity (GB)	Case	Capacity (GB)
Bagel labs	Morphotype data	206,000	0.02	167,000	0.04
	Morphotype analysis data	298,000	0.03	247,000	0.08
Huray Positive	Self-recorded data	664,130	0.01	301,988	<0.01
	Intervention data	2781	<0.01	1769	<0.01
Goodoc	Medical service data	6,562,939	1.37	11,642,068	2.10
	Registry service data	8,170,880	0.64	10,722,939	1.73
	Medical consulting data	7,034,037	0.64	11,632,131	1.86
	Insurance service data	105	<0.01	115	<0.01
	Vaccination	-	-	3953	0.02
K-weather	Life-air data for house	76,039,210	0.37	222,370,000	2.39
	Life-air data for school	110,532,228	0.55	173,980,000	2.58
	Life-air data for crowd facilities	14,331,629	0.07	44,400,000	0.63
	Health environment index	432,960	0.01	1,050,000	0.05
	Lifelog data of a vulnerable social group	6,785,432	0.03	189,110,000	2.16
	Clinical trials in Wonju	-	-	370,530,000	4.11
I-SENS	Chronic disease analysis data	523,504	0.05	440,158	0.10
Healthmax	Metabolic syndrome’s data	11,207,155	0.82	4,794,343	4.14
LG U Plus *	Lifelog on communication	-	-	15,597,222	1.66
Health Bridge *	Lifelog under stress	-	-	9262	<0.01

* is the new data center in 2021.

**Table 4:** Defects ratio and Six-Sigma value in data validation.

Evaluation Factors	2020	2021
The number of opportunities	906,084,543	82,727,257,835
The number of defects	111,704	27,203,636
DPO	1.23 × 10^−4^	3.28 × 10^−4^
DPMO	123	329
Defects ratio	0.01%	0.03%
Data consistency	99.99%	99.70
Six-Sigma	5.17	4.91

**Table 5:** The result of de-identification.

Parameters	Description	Records at Risk(%)		Highest Risk(%)		Success Risk(%)		De-Identification Method
		Before	After	Before	After	Before	After	
WNJU_BLOD_ID	Patient ID	-	-	-	-	-	-	Encryption
INDVDL_FLNM	Patient name	-	-	-	-	-	-	Remove
BRDT	Birthday	100	0	100	0.51	100	0.51	Masking
ADDR	Address	100	0	100	4	100	4	Masking
MBL_NO	Mobile	100	0	100	1.58	100	1.02	Masking
AGE	Age	15.30	0	100	5.23	16.83	3.06	Interval
TC	Total cholesterol	94.89	0	100	5.26	53.57	1.53	Interval
ALBMN	Albumin	100	0	100	5.32	92.34	1.57	Interval
AST	AST	17.85	0	100	<0.1	19.89	<0.1	Interval
ALT	ALT	40	0	100	0.22	27.04	<0.1	Interval
GGTP	γ-GTP	100	0	100	0.51	97.95	0.51	Interval
LDL	LDL	97.44	0	100	0.51	53.06	0.51	Interval
HDL	HDL	30.61	0	100	20	23.97	2.04	Interval
Cr	Creatin	100	0	100	8.33	97.96	2.55	Interval
BUN	Blood urea nitrogen	86.73	0	100	16.66	53.06	1.02	Interval
WBC	White blood cell count	100	1.53	100	33.33	98.46	4.08	Interval
PLT	Platelet	100	0	100	20	69.38	1.53	Interval

**Table 6:** The list of developed APIs.

API Name	Description	URL	Example of the Output
ckan.logic.action.create.package_create (POST)	Creation of packages	http://platform domain:8080/api/action/package_create	{ "help": "http://API url", "success": true, {"author": , … "creator_user_id":"2f53c018-…-8f9d-1875", "isopen": false, "license_id": "version": null, "extras": [ { "key": "paid_gb", "value": "1" } ], … }
ckan.logic.action.get.package_search (GET)	Searching for data list and information	http://platform domain:8080/api/action/package_search	{ "help": "http://API url", "success": {"author": "yj", … "owner_org": "19f75d75- … -9df8-7231bf67", "period": "yearly", "prodCode": "LI03090002", "species_cd": "LI03200009", "state": "active", … }
ckan.logic.action.get.package_show (GET)	Information of the specific package	http://platform domain:8080/api/action/package_show	{ "help": "http://API url", "success": {"author": "yj", … "tags": [{ "display_name": "tag_name", "id": "bf71a7ce-a6bf-443d-9d17-3ad2c9d7b3", "name": "tag", "state": "active", "vocabulary_id": null … }
ckan.logic.action.create.package_patch (POST)	Updating the information of the specific package	http://platform domain:8080/api/action/package_patch	{ "help": "http://API url", "success": {"author": "yj", … "prodCode": "LI032000090002", "species_cd": "LI03200009", "state": "active", "title": "update the title", "type": "dataset", … }
ckan.logic.action.delete.package_delete (POST)	Deletion of the package	http://platform domain:8080/api/action/package_delete	{ "help": "http://API url", "success": true, “result”:null }
ckan.logic.action.create.resource_create (POST)	Registration of package	http://platform domain:8080/api/action/resource_create	{"help":"url": http://API url:8080/dataset/ 88b37228-b57c-46b5-9eaf-e4d256985a4b/ resource/fe49dfba-3f20-43fc-…4762618 /download/iris.csv … }
ckan.logic.action.patch.resource_patch (POST)	Updating meta information of attached files in the package	http://platform domain:8080/api/action/resource_patch	{ "help": "http://API url", "success": true, {"author": , … "mimetype": "text/csv", "mimetype_inner": null, "name": "resource name", "package_id":"88b37228- ... -e4d256985a4b", … }
ckan.logic.action.delete.resource_delete (POST)	Deletion of the file in the package	http://platform domain:8080/api/action/resource_create	{ "help": "http://API url", "success": true, “result”:null }
ckan.logic.action.get.statistics_list (GET)	Retrieval of the statistic by organizations and resources	http://platform domain:8080/api/action/statistics_list	{ "help": "http://API url", "success": true, {"author": , … "results": [{ "title": "YWMC", "resoures_count": 136, "package_count": 58, "name": "yonseuniv", "free": 0, "pay": 58, "format": { "CSV": 99, "ZIP": 37} … }
ckan.logic.action.create.schema_create (POST)	Registration of the schema of the package	http://platform domain:8080/api/action/schema_create	{ "help": "http://API url?name=schema_create","success": true, "result": { "success": "data insert success" } }
ckan.logic.action.get.schema_search (GET)	Retrieval of the schema of the package	http://platform domain:8080/api/action/schema_search	{ "help": "http://API url", "success": true, {"author": … "result": { "prodCode": "LI091050111113", "columns": [{"seq": 1,"name": "DTM_AQ ", "data_type": "text", "max_length": 100, … }
ckan.logic.action.get.schema_delete (POST)	Deletion of the schema of the package	http://platform domain:8080/api/action/schema_delete	{ "help": "http://API url?name=schema_delete","success": true, "result": { "success": "data delete success" } }
ckan.logic.action.create.species_create (POST)	Registration of data items	http://platform domain:8080/api/action/species_create	{ "help": "http://API url?name=schema_create","success": true, "result": { "success": "LI012000025" } }
ckan.logic.action.get.species_list (GET)	Retrieval of data items	http://platform domain:8080/api/action/species_list	{ "help": "http://API url", "success": true, {"author": … "result": {"count": 143,"result": [{"species_cd": "LI10200001", "prodcode": "LI10200010011", "metadata_modified":"2021-07-28T06:02:34.015405" … }
ckan.logic.action.patch.species_patch (POST)	Updating the information of the data item	http://platform domain:8080/api/action/species_patch	{ "help": "http://API url?name=schema_patch", "success": true, "result": { "success": "data patch success" } }
ckan.logic.action.delete.species_delete (POST)	Deletion of the data item	http://platform domain:8080/api/action/species_delete	{ "help": "http://API url?name=speices_delete","success": true, "result": { "success": "delete success" } }
ckan.logic.action.get.organization_list (GET)	Retrieval of the organization list	http://platform domain:8080/api/action/organization_list	{ "help": "http://API url", "success": true, {"author":, … "result": ["ywmc", "hallymuniv"…, “koreauniv”] }

## Data Availability

Not applicable.
